# THE VALUE OF IN-PERSON VA MEDICAL FOSTER HOME NATIONAL CONFERENCE FOR RURAL PROGRAM COORDINATORS

**DOI:** 10.1093/geroni/igae098.4093

**Published:** 2024-12-31

**Authors:** Chelsea Manheim, Rachel Fix, Leah Haverhals

**Affiliations:** Rocky Mountain Regional VA Medical Center, Aurora, Colorado, United States; Rocky Mountain Regional VA Medical Center, Aurora, Colorado, United States; Rocky Mountain Regional VA Medical Center, Aurora, Colorado, United States

## Abstract

The Department of Veterans Affairs (VA) Medical Foster Home (MFH) program provides long-term care for nursing home eligible Veterans who desire to reside in home-like environments. In MFHs, Veterans are matched with non-VA caregivers to live in caregivers’ homes as part of their family, with caregivers providing around-the-clock care for up to three patients. MFH coordinators are responsible for full program implementation including recruiting, screening, and monitoring caregivers; recruiting Veterans; and partnering with VA’s Home Based Primary Care teams who provide Veterans’ healthcare in MFHs. Balancing these many duties, the MFH coordinator position can prove isolating. In 2024, the VA Office of Geriatrics and Extended Care (GEC) sponsored two, two-day, conferences for MFH staff to encourage goal setting, cross-site collaboration, and foster support. The first conference hosted 83 attendees from east coast programs in Orlando, Florida in March, and the second hosted 98 attendees from Midwest and western programs in Chicago, Illinois in May. Conferences coincided with VA efforts to expand MFH programs to all VA Medical Centers (VAMCs) by 2026. Additionally, the VA Office of Rural Health (ORH) currently funds expansion of four rural MFH programs. To assess experiences of MFH coordinators from the four rural expansion programs, we conducted interviews with them in June 2024. Coordinators reported making strong connections with more seasoned coordinators in nearby areas, developed innovative marketing ideas and created action plans, and improved motivation to serve in rural areas. Interviews showed that in-person conferences provide needed boosts to coordinators working in solitary positions.

